# Primary infection with a Brazilian chikungunya virus isolate protects immunocompetent mice against detectable viremia following homologous rechallenge

**DOI:** 10.1007/s00705-026-06724-z

**Published:** 2026-08-20

**Authors:** Naiara Soares da Silva, Mayanna Moreira Costa Fogaça, Elmo José dos Santos, Bruna Santos Souza da Silva, Ruth Dálety da Silva Brito, Rodrigo Lucas Ferreira do Carmo, Amanda da Rocha Santos, Milena Silva Souza, Tarcisio Macedo Silva, Eric Roberto Guimarães Rocha Aguiar, Alexander Birbrair, Paloma Oliveira Vidal, Jaime Henrique Amorim

**Affiliations:** 1 Center of Biological Sciences and Health, Western Bahia Virology Institute, Federal University of Western Bahia, Barreiras, Bahia Brazil; 2 Department of Biological Sciences, State University of Santa Cruz, Ilhéus, BA Brazil; 3 Virus Bioinformatics Laboratory, Biological Sciences Institute, Federal University of Minas Gerais, Minas Gerais, Belo Horizonte, MG Brazil; 4 Department of Dermatology, School of Medicine and Public Health, University of Wisconsin-Madison, Madison, WI USA; 5 Western Bahia Virology Institute, Center of Biological Sciences and Health, Federal University of Western Bahia, Rua da Prainha, Morada Nobre, Barreiras, Bahia 1326 Brazil

## Abstract

Chikungunya virus (CHIKV) is an arthropod-borne virus responsible for an acute febrile illness characterized by severe polyarthralgia, predominantly affecting peripheral joints. In a substantial proportion of patients, these symptoms may progress to chronic manifestations that significantly impair quality of life. Animal models are therefore critical for the development of effective therapeutic and preventive strategies, including antiviral agents and vaccines. In this context, the aim of this study was to establish an experimental model of CHIKV infection using immunocompetent C57BL/6 mice. Animals were infected with CHIKV and subsequently monitored for clinical signs, viremia, viral dissemination across tissues, total leukocyte counts, and susceptibility to reinfection. Results revealed no deaths among the animals during the experimental period, and infected animals exhibited significant viremia as well as detectable viral loads in the popliteal lymph node, femoral bone marrow, and muscle, demonstrating CHIKV capacity to disseminate and reach multiple target tissues. In addition, a global reduction in leukocyte counts was observed in infected mice. Notably, animals previously infected with CHIKV were resistant to reinfection, indicating the establishment of a protective immune response, as evidenced by the presence of neutralizing antibodies. In conclusion, the murine model established in this study recapitulates key features of CHIKV infection and provides a robust platform for investigating viral pathogenesis, as well as for evaluating novel antiviral strategies and vaccine candidates.

**Supplementary Information:**

The online version contains supplementary material available at https://doi.org/10.1007/s00705-026-06724-z.

## Introduction

Chikungunya virus (species *Alphavirus chikungunya*, CHIKV), within the genus *Alphavirus* and family *Togaviridae*, is an enveloped positive-sense single-stranded RNA virus transmitted mainly by *Aedes aegypti* and *Aedes albopictus* mosquitoes [1]. Since its first isolation during an outbreak in Tanzania in 1953, CHIKV has emerged as one of the most important arthropod-borne viruses affecting human populations worldwide [2]. The viral genome is approximately 11.8 kb in length and encodes four nonstructural proteins involved in viral replication and five structural proteins, including the E1 and E2 envelope glycoproteins, which play essential roles in viral attachment, membrane fusion, and host cell entry [2]. Due to the high mutation rates characteristic of RNA viruses, CHIKV has diversified into distinct genotypes [2, 3], including the West African (WA), East/Central/South African (ECSA), and Asian genotypes, with the Indian Ocean lineage (IOL) emerging as a derivative of the ECSA genotype associated with large outbreaks and adaptive mutations linked to vector competence.

Over the last two decades, CHIKV has expanded across multiple continents, causing relevant epidemics in Africa, Asia, Europe, and the Americas [2, 4]. The introduction of CHIKV into the Americas in 2013 represented a major public health milestone, followed by rapid dissemination throughout tropical and subtropical regions [2, 5]. In Brazil, the first autochthonous cases were reported in 2014, and since then the virus has become firmly established, with recurrent epidemic waves and widespread territorial distribution across all Brazilian regions [6–8]. Brazil rapidly became one of the global epicenters of chikungunya transmission, accounting for a substantial proportion of cases reported in the Americas. Molecular epidemiological studies demonstrated the predominance of the ECSA genotype in several Brazilian regions, including Bahia, where intense viral circulation has been documented in recent years [8]. The combination of high vector density, urbanization, climatic conditions favorable to mosquito proliferation, and population susceptibility contributed to sustained viral transmission and recurrent outbreaks in the country.

Clinically, CHIKV infection is characterized by an acute febrile illness frequently accompanied by severe polyarthralgia, myalgia, headache, fatigue, and rash [2, 3]. During the acute phase, patients develop significant viremia, which contributes to systemic dissemination of the virus to multiple tissues, including joints, muscle, lymphoid organs, and, in some cases, the nervous system. CHIKV pathogenesis is strongly associated with intense inflammatory responses driven by innate and adaptive immune activation, including the production of proinflammatory cytokines and chemokines. Although most patients recover from the acute infection, a substantial proportion develop chronic arthralgia and persistent musculoskeletal manifestations that can last for months or even years, significantly impacting quality of life [3, 4]. Understanding the mechanisms involved in viral dissemination, tissue tropism, immune responses, and protective immunity remains essential for the development of antiviral therapies and vaccines. In this context, experimental animal models are critical tools for investigating CHIKV pathogenesis and evaluating candidate interventions under controlled conditions.

Several murine models have been developed to study CHIKV infection, including neonatal mice, interferon receptor-deficient animals, and immunocompetent strains such as C57BL/6 mice [9, 10]. However, many currently available models rely on immunodeficient animals, or experimental conditions that do not fully reproduce important aspects of natural human infection. In addition, relatively few studies have evaluated animal infection using recent Brazilian clinical isolates representing viruses currently circulating during epidemic waves in the country. Therefore, the development of reproducible experimental models employing clinically relevant isolates may contribute to a more accurate understanding of viral pathogenesis, tissue dissemination, viremia kinetics, and protective immune responses. In the present study, we established and characterized an experimental CHIKV infection model using immunocompetent C57BL/6 mice infected with a Brazilian clinical isolate obtained during the 2021 outbreak in western Bahia, aiming to advance available experimental platforms and provide a model that more closely reflects contemporary CHIKV epidemiology and infection dynamics.

## Materials and methods

### Virus

The CHIKV35 strain used in this study was isolated from a human serum sample collected in 2021 in Barreiras, western Bahia [8]. The isolate identity was confirmed by RT-qPCR and sequencing EPI_ISL_20448531. The virus was propagated three times in C6/36 cells to generate a working stock. All procedures involving human samples were approved by the Research Ethics Committee (CAAE 68131723.2.0000.8060).

### Animal model

**Fig. 1:**
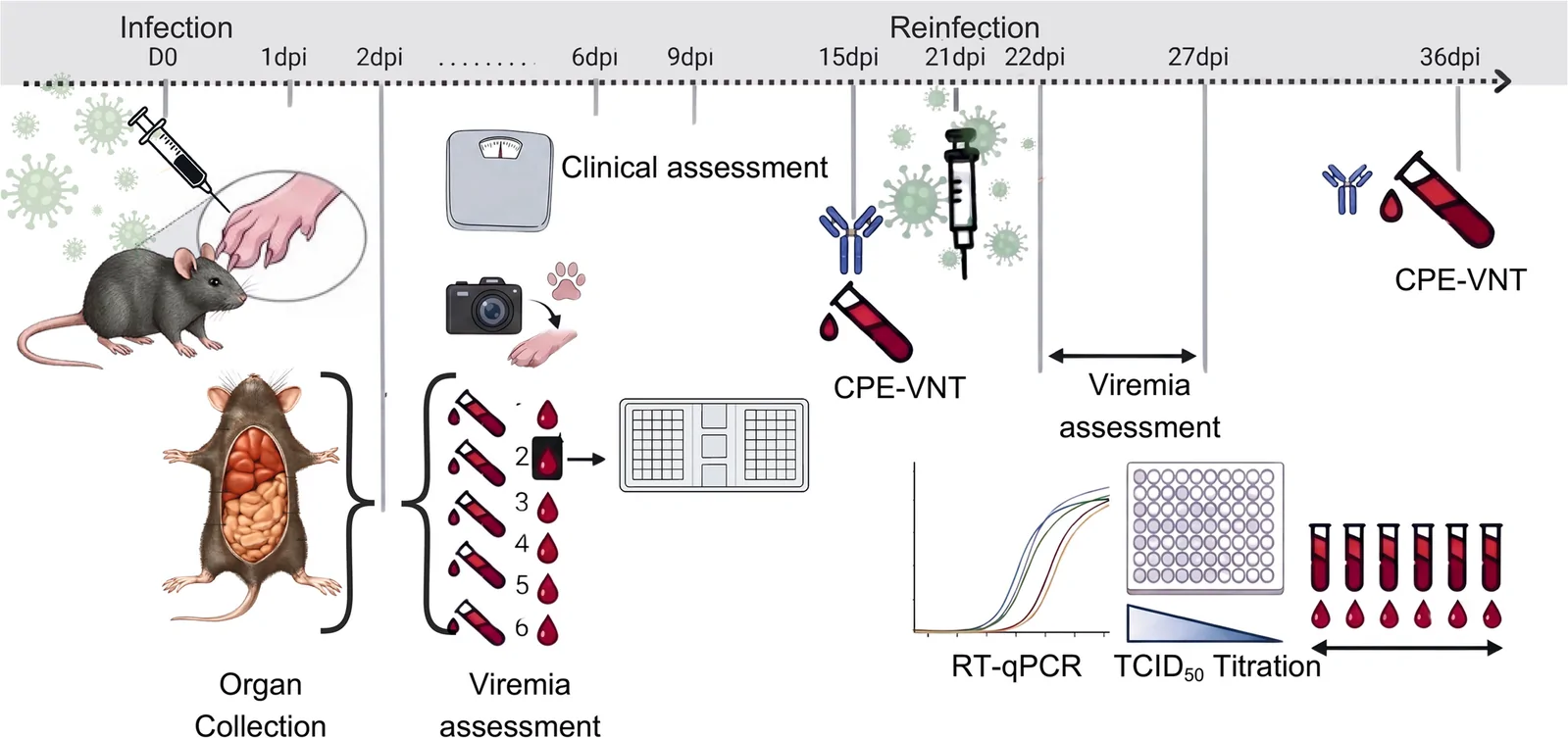
Experimental design of the CHIKV infection and reinfection model in immunocompetent C57BL/6 mice. Male and female C57BL/6 mice (8 weeks old) were inoculated subcutaneously in the left hind footpad on day 0 with 50 µL of the CHIKV35 strain (1.58 × 10^5^ TCID₅₀). Peripheral blood samples were collected daily from 1 to 6 days post-infection (dpi) for viremia assessment by RT-qPCR and TCID₅₀ assays. The remaining animals (*n* = 10 per group) were monitored longitudinally throughout the experiment. Clinical parameters, including body weight variation, leukocyte counts, and paw edema, were monitored throughout the acute phase of infection. On day 15 dpi, serum samples were collected for the detection of neutralizing antibodies by CPE-VNT. On day 21 dpi, animals from both infected and control groups were challenged again with the same CHIKV35 inoculum. Following reinfection, peripheral blood samples were collected from 21 to 27 dpi for secondary viremia analysis. Final serum samples were obtained on day 36 dpi for evaluation of neutralizing antibody responses after reinfection. Schematic elements were created to summarize the longitudinal experimental workflow used in this study. The figure was partially developed using generative artificial intelligence tools integrated into Canva Pro

The animals were allocated into two experimental groups: infected group and control group. The infected group (*n* = 14) was inoculated subcutaneously (s.c.) in the left hind footpad with 50 µL of the CHIKV35 strain, corresponding to 1.58 × 10^5^ TCID₅₀, similarly as previously described [11].To assess viremia, peripheral blood was collected daily for 6 days, and total leukocyte counts were performed using a Neubauer chamber, as previously described [12]. Animals in the control group (*n* = 14) received 50 µL of DMEM only via s.c. injection in the left hind footpad. Two male and two female mice from each group (control and infected) were euthanized on day 2 post-infection (dpi) for tissue collection and viral dissemination analyses. Spleen, liver, kidneys, brain, popliteal lymph node, thigh muscle, and femoral bone marrow were collected, and individually homogenized in a fixed volume of 1 mL sterile DMEM using a class II, type A2 biosafety cabinet (Lutech, Brazil). After centrifugation, the resulting supernatants were subjected to RNA extraction and analyzed by RT-qPCR for viral detection and quantification, followed by TCID₅₀ titration. Viral loads were expressed as TCID₅₀/mL and eTCID₅₀/mL of tissue homogenate supernatant. The remaining animals (*n* = 10 per group) were followed longitudinally for analyses including body weight variation, leukocyte counts, paw edema assessment, molecular and infectious viremia, and neutralizing antibody responses. Body weight variation was calculated daily for each animal up to 9 dpi and expressed as the percentage of final weight compared to initial weight [12]. Additionally, the inoculation site was monitored daily and documented through photographic records to track swelling development resulting from infection. On days 15 and 36 dpi, blood samples were collected for neutralizing antibody titration using the cytopathic effect-based virus neutralization test (CPE-VNT) method. On day 21 dpi, mice from both experimental groups were subjected to homologous rechallenge with CHIKV35. In this procedure, animals from infected and control groups were s.c inoculated in the left hind footpad with 50 µL of the CHIKV35 strain, corresponding to 1.58 × 10^5^ TCID₅₀. Peripheral blood was again collected daily up to 6 days post-reinfection to assess viremia. Due to limitations in blood volume collection during some time points, not all analyses included samples from all 10 animals, and therefore certain datasets may represent fewer animals (see Fig. 1).

### Ethics statement

All experimental procedures were approved by the Animal Ethics Committee of the Federal University of Western Bahia (CEUA/UFOB, 0015-2024) and conducted in accordance with the guidelines for the care and use of laboratory animals of the National Council for the Control of Animal Experimentation, CONCEA, Brazil. Male and female C57BL/6 mice, aged 8 weeks, were used in this study. The mice were obtained from the animal facility at the Federal University of Western Bahia and maintained under controlled conditions (23.4 ± 0.63 °C, 12-h light/dark cycle), with water and food provided *ad libitum*.

### Cell culture and virus stock

C6/36 and Vero CCL-81 cell lines were kindly provided by Dr. Carla Torres Braconi (Federal University of São Paulo, Brazil). Both cell lines were used at low to moderate passages and maintained under stable culture conditions throughout the experiments. Cell cultures were routinely examined by microscopy to ensure morphological integrity and check for contamination. C6/36 cells were cultured in Leibovitz L-15 medium supplemented with 10% fetal bovine serum (FBS) and 1% penicillin-streptomycin (Thermo Fisher Scientific, USA) at 27 °C in a controlled atmosphere. Vero CCL-81 cells were maintained in DMEM medium containing 10% FBS and 1% penicillin-streptomycin (Thermo Fisher Scientific, USA) at 37 °C in a 5% CO₂ atmosphere. The CHIKV35 strain was propagated in C6/36 cells. For working stock preparation, C6/36 cell monolayers at up to 70% confluence were inoculated with 50 µL of the viral strain in a final adsorption volume of 1 mL. Adsorption occurred over 1 h at 27 °C, with gentle rocking every 15 min. Adsorption supernatants were then aspirated and replaced with L-15 medium supplemented with 2% (v/v) FBS and 1% (v/v) antibiotics. Viral cultures were monitored daily by optical microscopy for 72 h. Detection and absolute or relative quantification of CHIKV35 were performed by RT-qPCR (reverse transcription followed by real-time polymerase chain reaction). After propagation, the viral stock was concentrated by 50% polyethylene glycol (PEG) precipitation. Propagation supernatants were transferred to 50-mL conical tubes and centrifuged at 3,200 × g for 15 min at 4 °C. The resulting pellets, containing residual supernatant volume, underwent three freeze-thaw cycles (-80 °C for 5 min; water bath at 37 °C for 2 min). Pellets were then centrifuged at 3,200 × g for 15 min at 4 °C, and the recovered supernatants were pooled with the initial reserved supernatants. Subsequently, 50% polyethylene glycol (PEG 6000) (Synth, Switzerland) was added, and suspensions were incubated overnight at 4 °C. The next day, suspensions were centrifuged at 3,200 × g for 30 min at 4 °C. Supernatants were discarded, and viral precipitates were resuspended in DMEM + HEPES buffer (4-(2-hydroxyethyl)-1-piperazineethanesulfonic acid, HEPES 10 mM, NaCl 150 mM, KCl 5 mM, Na₂HPO₄ 1 mM, pH 7.2) at a ratio of 100 µL HEPES per 10 mL of original culture. Stocks were aliquoted and stored at − 80 °C until use in subsequent experiments.

### Viral titration by TCID₅₀ assay

The viral titration assay was adapted from the protocol described by dos Santos et al. [13]. For replication kinetics, four independent biological replicates were used. Briefly, Vero CCL-81 cells were seeded in 96-well plates (1 × 10^4^ cells/well) and incubated overnight under standard culture conditions to allow cell adhesion. The following day, CHIKV35 stock inoculum, serum samples or organ supernatant samples were subjected to 10-fold serial dilutions (10^− 1^ to 10^− 12^) in DMEM culture medium supplemented with 1% (v/v) FBS and 1% (v/v) antibiotics. One hundred microliters of each dilution were added to four wells containing the respective cell monolayers. After a 1-hour incubation period to allow viral adsorption, the inoculum was removed and replaced with 100 µL of fresh DMEM supplemented with 2% FBS and 1% antibiotics. Plates were incubated for 72 h at 37 °C in a 5% CO₂ atmosphere. Cytopathic effect (CPE) was monitored daily using an inverted light microscope. After incubation, cells were washed with PBS, fixed with 1% (v/v) formaldehyde for 30 min, and stained with 5% crystal violet solution for 15 min. The 50% tissue culture infectious dose (TCID_50_) was calculated using the Reed-Muench method, considering the presence or absence of CPE in each replicate.

### Nucleic acid extraction and RT-qPCR

RNA was extracted from CHIKV35-infected cell culture supernatants, serum samples collected from infected and control C57BL/6 mice, and supernatants obtained from macerated organ suspensions using the Loccus Viral RNA/DNA Extraction Kit, processed on the automated Extracta32 platform (Loccus, Brazil), according to the manufacturer’s instructions. Viral quantification and confirmation were performed by real-time reverse transcription polymerase chain reaction (RT-qPCR) using the QIAquant thermocycler (Qiagen, Germany). CHIKV detection was carried out using the VIASURE Zika, Dengue & Chikungunya Real Time PCR Detection Kit (CerTest Biotec, Spain), following the manufacturer’s instructions. RT-qPCR reactions were performed in a final volume of 10 µL, consisting of 7.5 µL master mix and 2.5 µL extracted RNA.

### RT-qPCR standard curve

To generate the RT-qPCR standard curve, viral RNA was extracted from the CHIKV35 strain, which had been previously titrated using the TCID₅₀ assay in Vero CCL-81 cells. The resulting TCID₅₀ value was used as the reference for equivalent viral quantification by RT-qPCR. The purified RNA was serially diluted in tenfold steps (10^− 1^ to 10⁻⁷) using nuclease-free water. Each dilution point was prepared in duplicate to ensure reproducibility. RT-qPCR reactions were performed using the VIASURE Zika, Dengue & Chikungunya Real Time PCR Detection Kit (CerTest Biotec, Spain), following the manufacturer’s instructions. RT-qPCR reactions were performed in a final volume of 10 µL, consisting of 7.5 µL master mix and 2.5 µL extracted RNA. Reactions were carried out on a QIAquant real-time PCR system (Qiagen, Germany). Cycle threshold (Ct) values obtained for each dilution were used to construct the standard curve. Reaction efficiency was calculated from the slope of the curve, with acceptable parameters defined as a correlation coefficient (R²) ≥ 0.98 and amplification efficiency between 90% and 100%.

### Viral replication kinetics

Four culture flasks containing confluent monolayers of Vero cells were infected with CHIKV35 at a multiplicity of infection (MOI) of 0.01, following the infection protocol described previously [13]. After viral adsorption and replacement with incubation medium (DMEM supplemented with 2% FBS and 1% antibiotics), 200 µL aliquots of culture supernatant were collected at 0, 1, 3, 6, 12, 24, 48, 72, and 96 h post-infection. Each aliquot was immediately stored at − 80 °C for later analysis. Stored samples were subsequently processed for TCID_50_.

### Cytopathic effect-based virus neutralization test

The CPE-VNT was adapted from the protocol described by dos Santos et al. [13]. Initially, Vero CCL-81 cells were cultured in sterile 96-well plates to reach 1 × 10^5^ cells per well. Serum samples collected from the animals were heat-inactivated to complement inactivation by incubation at 56 °C for 30 min. Samples were serially diluted in log_2_ steps, with a dilution range from 1:20 to 1:1,280. Diluted samples were incubated with equal volumes of 1 × 10^3^ TCID_50_ CHIKV35 strain at 37 °C for 1 h. Subsequently, the mixtures were added to pre-designated culture plates to allow viral adsorption at a MOI of 0.01, for 1 h at 37 °C in a 5% CO₂. After this period, neutralization mixture supernatants were removed and replaced with DMEM culture medium containing 2% (v/v) FBS and 1% (v/v) antibiotics, followed by incubation for 48 h. CPE was examined under a microscope, after which cells were fixed with 10% formaldehyde solution and stained with 5% crystal violet solution.

### Virus genome sequencing and phylogeny

The complete genome of the CHIKV35 strain, isolated from a human serum sample collected in 2021 in Barreiras, western Bahia, Brazil [8], was obtained by nanopore sequencing. Viral RNA was initially reverse transcribed into complementary DNA (cDNA) using the LunaScript^®^ RT SuperMix Kit (New England Biolabs, Massachusetts, USA), according to the manufacturer’s instructions. Subsequently, whole-genome amplification was performed by conventional PCR (cPCR) using a previously described tiling primer scheme for CHIKV genome amplification and the Q5^®^ High-Fidelity DNA Polymerase (New England Biolabs, Massachusetts, USA), following the manufacturer’s recommendations. PCR reactions were prepared using Q5 reaction buffer (1× final concentration), primer pools at a final concentration of 0.015 µM per primer, ultrapure water, and 2.5 µL of cDNA template. Following amplification, sequencing libraries were prepared using the Native Barcoding Kit 96 V14 (SQK-NBD114.96, Oxford Nanopore Technologies, United Kingdom), according to the manufacturer’s instructions, and sequencing was performed on the MinION platform (Oxford Nanopore Technologies, United Kingdom). Raw reads were processed to generate a consensus genome sequence, which was subsequently aligned using Molecular Evolutionary Genetics Analysis (MEGA) version 11.0. For phylogenetic analyses, the complete genome sequence obtained for CHIKV35 was compared with representative CHIKV sequences belonging to the West African (WA), East/Central/South African (ECSA), and Asian genotypes retrieved from the GISAID database. The dataset included the following sequences: WA/EPI_ISL_18498036, WA/EPI_ISL_18498015, WA/EPI_ISL_18498019, WA/EPI_ISL_18498019(2), WA/EPI_ISL_19557253, WA/EPI_ISL_19557249, Asian/EPI_ISL_19086665, Asian/EPI_ISL_17498951, Asian/EPI_ISL_17485555, Asian/EPI_ISL_16554084, ECSA/EPI_ISL_17680374, ECSA/EPI_ISL_19772201, ECSA/EPI_ISL_20244567, ECSA/EPI_ISL_19411215, ECSA/EPI_ISL_20362540, and ECSA/EPI_ISL_20402648. Additionally, the RefSeq sequence NC_004162.2 (Chikungunya virus reference genome) and NC_001512.1 (O’nyong-nyong virus), used as the outgroup, were included in the analysis. Multiple sequence alignments were generated using MEGA version 11.0, and phylogenetic reconstruction was performed using the maximum likelihood method with 1,000 bootstrap replicates.

### Statistical analyses

Statistical differences between groups across different time points were assessed using two-way repeated-measures ANOVA. This approach was chosen because the data were collected longitudinally from the same animals, thus representing repeated measures, allowing appropriate consideration of the intrinsic correlation among sequential observations. For analyses of total leukocyte counts and neutralization titers, the assumption of normality was not met due to the small sample size; therefore, the nonparametric Mann–Whitney test was applied. Longitudinal changes in neutralization titers within the infected group were evaluated using the Wilcoxon signed-rank test. Statistical significance was established at *p* ≤ 0.05.

## Results

### Phylogenetic characterization and replication kinetics of the CHIKV35 isolate

**Fig. 2:**
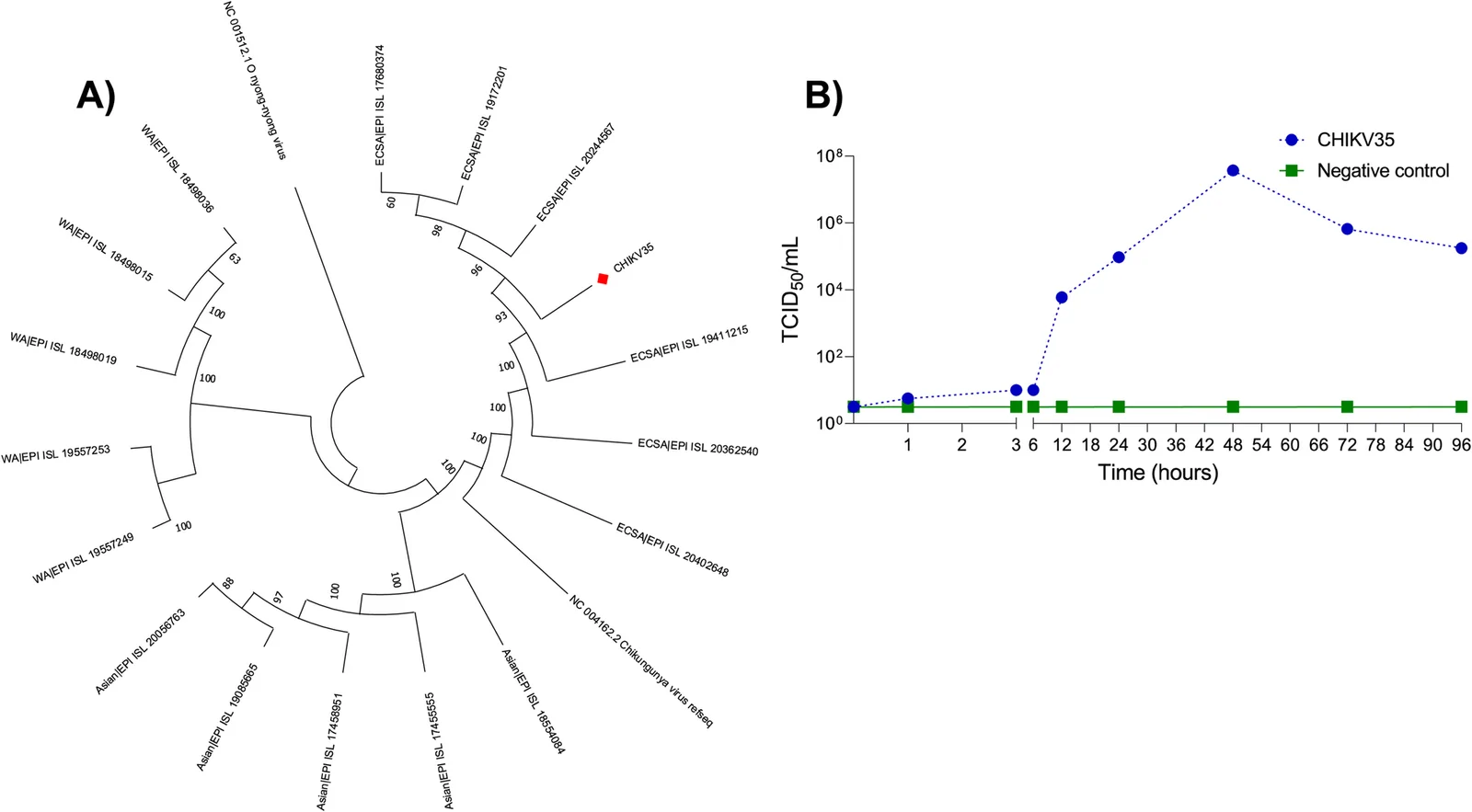
Phylogenetic characterization and replication kinetics of the CHIKV35. **A**, A maximum likelihood phylogenetic tree was constructed based on complete genome sequences of CHIKV35, as well as of CHIKV representative of WA, ECSA, and Asian genotypes. Bootstrap values (1,000 replicates) are indicated at the nodes. O’nyong‑nyong virus (ONNV) was used as the outgroup. **B**, viral growth kinetics of the CHIKV35 isolate in Vero CCL‑81 cells. Viral titers were expressed as TCID₅₀/mL

Phylogenetic analysis based on complete genome sequences demonstrated that the CHIKV35 isolate clustered within the East/Central/South African (ECSA) genotype, grouping closely with contemporary Brazilian strains (Fig. 2A). The topology obtained by the maximum likelihood method showed clear separation between the ECSA, West African (WA), and Asian genotypes, with strong bootstrap support across the major branches. O’nyong-nyong virus was used as the outgroup to root the phylogenetic tree. These findings confirmed the classification of CHIKV35 within the ECSA lineage currently circulating in Brazil.

The replication kinetics assay performed in Vero CCL-81 cells demonstrated progressive viral replication over time (Fig. 2B). Viral titers TCID₅₀/mL, increased markedly during the first 24 h post-infection, peaking at 48 h, followed by a gradual decline at later time points. These results indicate that the CHIKV35 isolate displays efficient replication capacity in mammalian cells under in vitro conditions.

### Clinical parameters following primary CHIKV35 infection in C57BL/6 mice

**Fig. 3:**
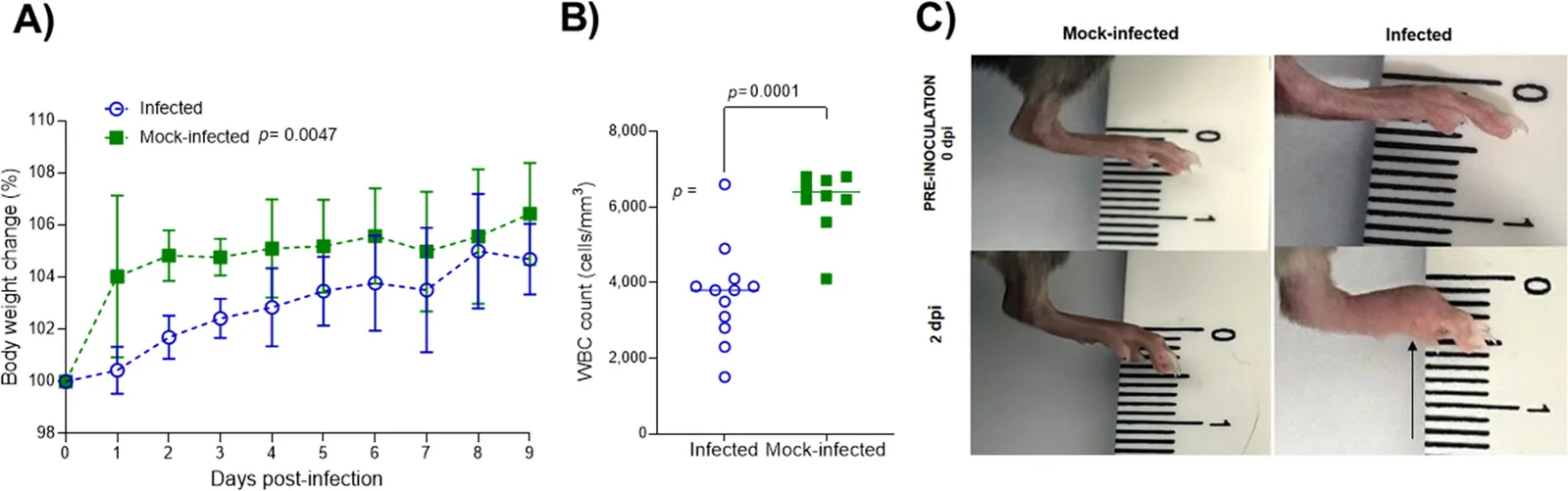
Clinical parameters following primary infection with CHIKV35 in immunocompetent C57BL/6 mice. **A**, body weight was monitored for up to nine dpi in infected (blue) and mock-infected (control, green) animals. Data are presented as percentage change relative to the initial body weight measured prior to injection. **B**, peripheral blood was collected at day 2 post-infection, and white blood cell (WBC) counts were determined using a Neubauer chamber. Results are expressed as cells/mm³. **C**, edema at the hind footpad was assessed by visual inspection before inoculation and at 2 days post-inoculation. Representative images from mock-infected and CHIKV-infected animals are shown before inoculation (upper panel) and at 2 dpi (lower panel). The arrow indicates the edematous area in the infected animal compared to the normal appearance in the control. Statistical significance was set at *p* ≤ 0.05. Data are representative of two independent experiments

Animals were subjected to clinical evaluation following primary infection with CHIKV35. Body weight monitoring showed that infected animals exhibited a significantly reduced body weight gain compared to mock-infected controls during the acute phase of infection (Fig. 3A). In addition, peripheral leukocyte counts evaluated at 2 days post-infection revealed a significant reduction in total white blood cells in infected animals compared to controls (Fig. 3B). This reduction was consistent with systemic viral infection and demonstrated an early hematological alteration associated with CHIKV35 infection. Relevantly, visual analysis of the inoculated hind footpad demonstrated evident edema in infected animals at day 2 post-infection, whereas mock-infected animals showed no visible alterations (Fig. 3C). Swelling was localized predominantly at the inoculation site and represented a clear inflammatory response induced by viral infection. Together, these findings indicate that CHIKV35 infection in immunocompetent C57BL/6 mice induced local inflammatory alterations and systemic leukocyte reduction in addition to causing significant effects on overall body weight during the acute phase. The survival rate was 100% in both experimental groups, no deaths were recorded during the experimental period.

### Viremia and tissue dissemination during primary infection

**Fig. 4:**
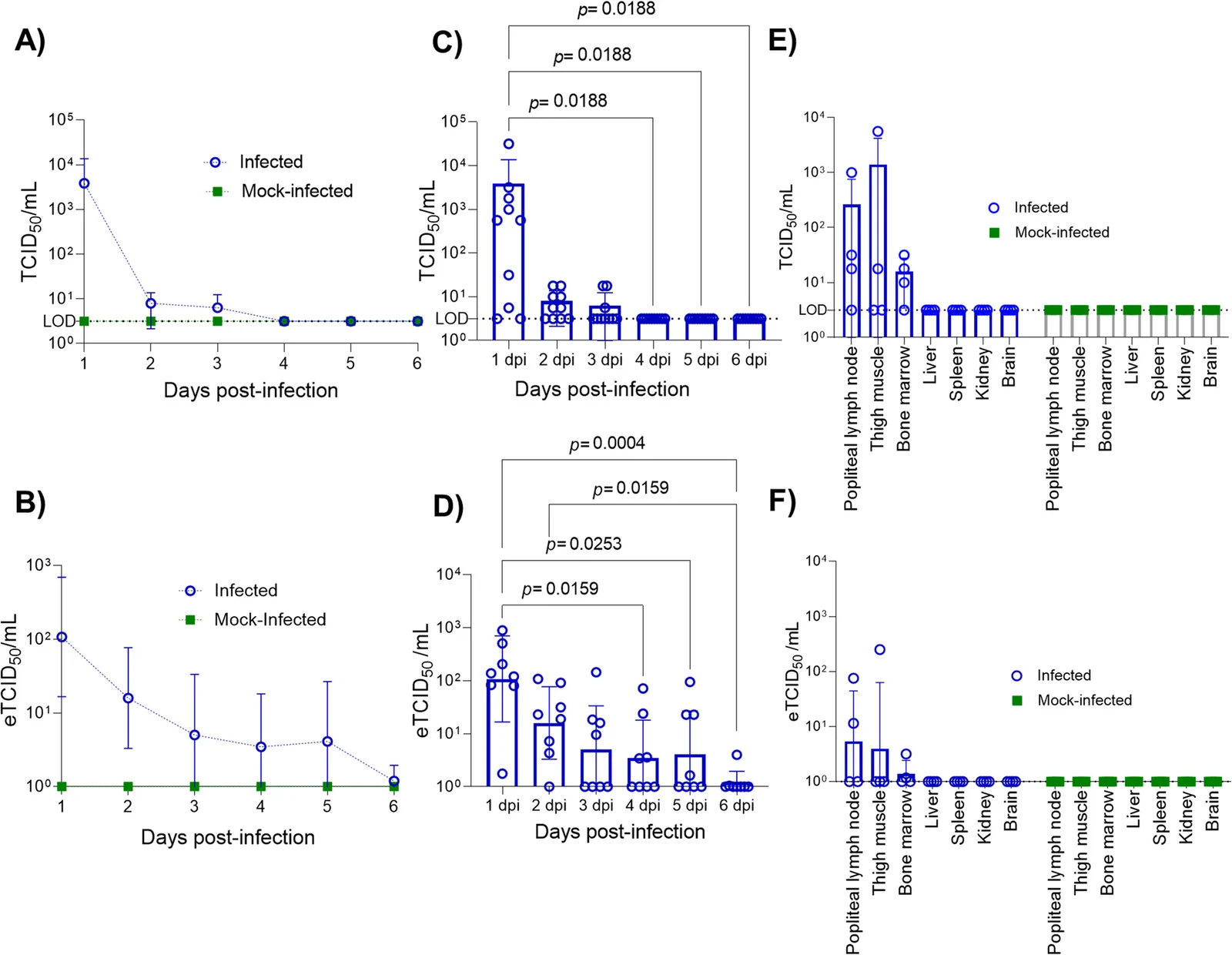
Detection and quantification of viremia in experimental groups. Peripheral blood samples were collected from control and infected animals and subjected to viral quantification by TCID₅₀/mL titration (**A**), as well as by estimated TCID₅₀/mL (eTCID₅₀/mL), calculated from Ct values obtained by RT-qPCR (**B**). Viral titers in blood samples from infected animals, measured up to 6 dpi by TCID₅₀ (**C**) or expressed as eTCID₅₀/mL (**D**), were statistically compared. Viral titers were determined as TCID₅₀/mL (**E**) or eTCID₅₀/mL (**F**) in organs from infected animals and statistically analyzed. Statistical significance was set at *p* ≤ 0.05. Data are representative of two independent experiments. The limit of detection (LOD) for the TCID_50_ assay was 3,16 × 10^0^ TCID_50_/mL

In addition to a clinical evaluation, viral load was monitored in experimental animal groups. Peripheral blood samples collected from infected animals demonstrated detectable viremia by both TCID₅₀ assay and RT-qPCR-derived equivalent TCID₅₀ quantification (Fig. 4A-B). No detectable virus was observed in mock-infected control animals. Longitudinal analysis of blood viral titers demonstrated that viremia was detectable during the first days following infection, with peak viral titers occurring during the early acute phase (Fig. 4C). Similar kinetics were observed when viral loads were estimated by RT-qPCR and expressed as eTCID₅₀/mL (Fig. 4D). The RT-qPCR quantification method was standardized using a reference curve presenting an R² of 0.9995 (Supplementary Fig. 1), confirming the consistency between molecular and infectivity-based quantification methods. In addition, analysis of tissue dissemination demonstrated the presence of infectious virus in selected organs collected from infected animals at 2 days post-infection (Fig. 4E-F). Viral detection was observed predominantly in the popliteal lymph node, muscle, and femoral bone marrow, whereas no detectable infectious virus or viral RNA were identified in the remaining analyzed organs. These findings demonstrate the ability of CHIKV35 to disseminate beyond the inoculation site and reach distinct target tissues and blood during acute infection. Overall, the results indicate that CHIKV35 establishes productive systemic infection in immunocompetent mice, characterized by clearly detectable viremia and dissemination to lymphoid and muscular tissues.

### Evaluation of viremia following secondary challenge

**Fig. 5:**
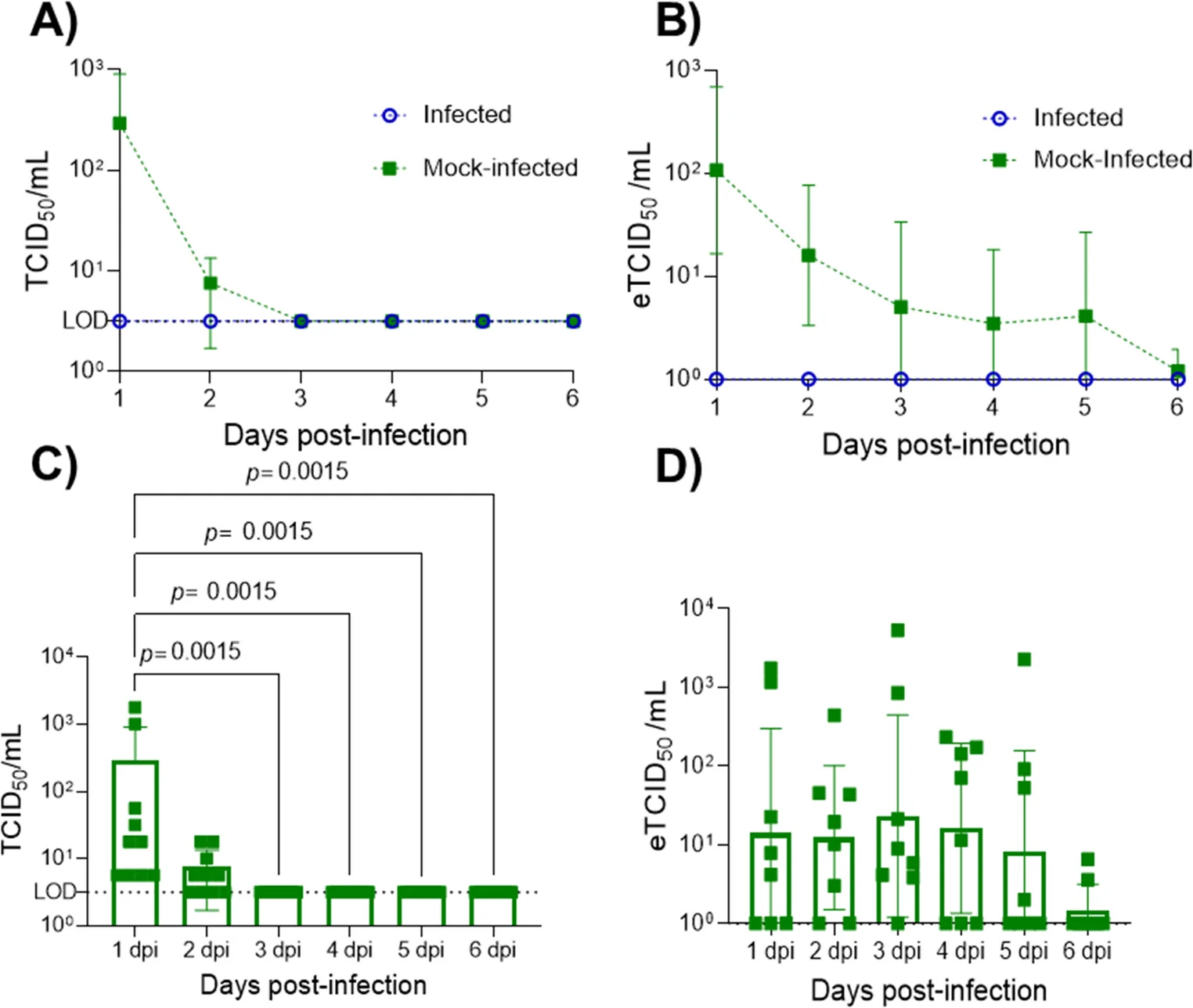
Detection and quantification of viremia in the experimental groups after secondary infection. Peripheral blood samples were collected in the first 6 days after injection from control and infected animals and subjected to viral quantification by TCID₅₀/mL titration (**A**), as well as by estimated TCID₅₀/mL (eTCID₅₀/mL) (**B**). Viral titers in blood samples from infected animals, measured up to 6 dpi by TCID₅₀ (**C**) or expressed as eTCID₅₀/mL (**D**), were statistically compared. Statistical significance was set at *p* ≤ 0.05. Data are representative of two independent experiments

Experimental animal groups were also subjected to a secondary infection. Following secondary challenge with CHIKV35, control animals that had not been previously exposed to the virus developed detectable viremia, as demonstrated by both TCID₅₀ assays and RT-qPCR-derived viral quantification (Fig. 5A-B). In contrast, previously infected animals showed absence of detectable viremia and no evidence of systemic viral replication. Longitudinal blood viral titers showed kinetics similar to those observed during primary infection, with productive infection detected exclusively in previously mock-infected animals. Similar findings were observed by both TCID₅₀ assay and RT-qPCR-derived equivalent TCID₅₀ quantification (Fig. 5C-D). These findings indicate that primary CHIKV35 infection induced a protective immune response capable of substantially restricting viral replication upon secondary exposure.

### Neutralizing antibody responses following primary infection and reinfection

**Fig. 6:**
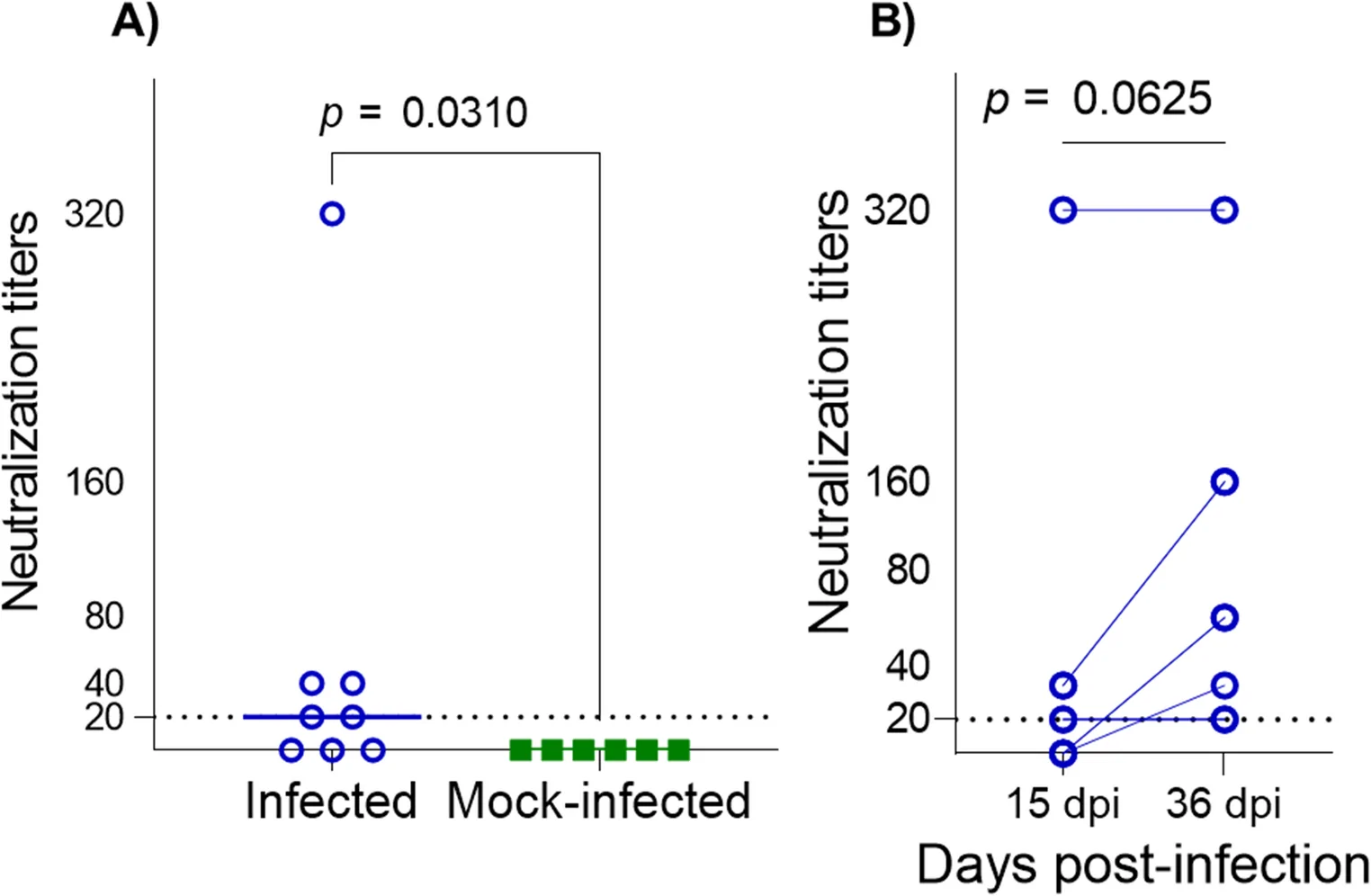
Analysis of neutralizing antibody titers following primary and secondary infection or mock infection. **A**, neutralizing antibody titers, assessed by CPE-VNT, were determined in serum samples collected 15 days post-injection and subjected to statistical analysis. **B**, neutralizing titers in infected animals were measured at 15 days after primary infection and at 15 days after secondary infection (corresponding to 36 days after primary infection). Data were statistically analyzed. Statistical significance was set at *p* ≤ 0.05. Data are representative of two independent experiments

Serum samples collected from animals of the two experimental groups in specific time points, both in primary and secondary infection, were subjected to CPE-VNT. As shown in Fig. 6A, detectable neutralizing responses were observed in infected animals at 15 days post-primary infection, whereas mock-infected animals showed no detectable neutralizing activity. Following secondary viral challenge, previously infected animals exhibited maintenance or enhancement of neutralizing antibody titers when evaluated at 36 days after primary infection (Fig. 6B). The increase in neutralizing activity after reinfection observed in some animals was consistent with the establishment of an adaptive humoral immune response induced during primary infection. Collectively, these findings indicate that CHIKV35 infection in immunocompetent C57BL/6 mice induces robust neutralizing antibody responses associated with protection against reinfection.

## Discussion

In the present study, we established and characterized an experimental model of chikungunya virus infection using immunocompetent C57BL/6 mice infected with a contemporary Brazilian CHIKV ECSA isolate. The model reproduced important virological and clinical aspects associated with acute CHIKV infection, including detectable viremia, local inflammatory manifestations, systemic hematological alterations, viral dissemination to target tissues, and the development of protective neutralizing immunity capable of preventing reinfection. Collectively, these findings support the utility of this model as a relevant experimental platform for studies investigating CHIKV pathogenesis, immune responses, antiviral therapies, and vaccine candidates.

One important aspect of the present study is the use of a Brazilian clinical isolate belonging to the ECSA genotype currently circulating during epidemic waves in the country. Since the introduction of CHIKV into Brazil, the ECSA genotype has become widely established and has been associated with sustained transmission and recurrent outbreaks in several regions, including Bahia [6, 8, 14–16]. Although several experimental CHIKV models have been established previously [10], the use of a contemporary Brazilian clinical isolate may improve the epidemiological relevance of the model and better represent currently circulating viral strains associated with recent outbreaks in the country. Phylogenetic analyses confirmed that the CHIKV35 isolate clustered with contemporary Brazilian ECSA strains, reinforcing the epidemiological relevance of the experimental model employed in this study.

The infection model induced detectable and productive systemic infection in immunocompetent animals, as demonstrated by both infectivity-based assays and molecular viral quantification. Importantly, the detection of viremia by TCID₅₀ and RT-qPCR-derived equivalent titration demonstrated strong agreement between methodologies, supporting the use of RT-qPCR-based approaches as reliable tools for monitoring viral kinetics in experimental settings. Peak viremia occurred during the early acute phase, similarly to what has been reported in both human infection and experimental mouse models of CHIKV disease [10, 17, 18]. These findings indicate that the CHIKV35 isolate displays efficient replication competence in vivo while maintaining a nonlethal infection profile compatible with longitudinal immune studies. In addition, although infected animals did not develop severe disease manifestations or mortality, clear clinical and inflammatory alterations were observed. Infected mice exhibited reduced body weight gain during the acute phase, accompanied by marked edema at the inoculation site and a reduction in peripheral leukocyte counts. Paw edema is a classical hallmark of CHIKV experimental infection and reflects local inflammatory responses induced by viral replication and immune activation within musculoskeletal tissues [10]. Similarly, leukopenia has been described in human chikungunya cases and may reflect systemic inflammatory responses and transient hematopoietic alterations during acute infection [19]. The observation that these alterations occurred in immunocompetent animals without severe neurological disease or lethality further supports the translational relevance of the model for studying mild-to-moderate CHIKV disease.

Beyond the systemic and local inflammatory alterations, the evaluation of viral tissue distribution provided additional insights into the dissemination profile of the CHIKV35 isolate in immunocompetent animals. Tissue dissemination analyses demonstrated the presence of virus predominantly in the popliteal lymph node, muscle, and femoral bone marrow during acute infection. These findings are consistent with the known tropism of CHIKV for musculoskeletal and lymphoid tissues and reinforce previous observations demonstrating viral dissemination beyond the inoculation site during systemic infection [20, 21]. Detection of virus in muscle tissue is particularly relevant considering the important role of musculoskeletal involvement in CHIKV-associated disease and chronic arthralgia [22].

Additionally, detection of virus in the femoral bone marrow may suggest transient infection of hematopoietic or stromal compartments, since cells within this organ are susceptible to viral infection and may have their functional properties compromised [23]. This finding therefore represents a plausible mechanism underlying the leukopenia observed in our study. The reproducible viremia and systemic viral dissemination observed in this model further support its potential applicability for evaluating antiviral strategies aimed at limiting CHIKV replication and tissue spread. However, it is important to note that tissue collection was conducted at a single time point (2 dpi) consequently, we cannot fully exclude the possibility of an early transient viral release or a later dissemination to peripheral tissues.

One of the most relevant findings of the present study was the demonstration that primary CHIKV infection induced effective protection against secondary challenge. To our knowledge, few studies have longitudinally demonstrated effective protection against homologous CHIKV rechallenge in immunocompetent mice, particularly using a contemporary Brazilian ECSA isolate. Animals previously infected with CHIKV35 showed complete absence of detectable viremia following reinfection, whereas naïve control animals developed productive infection after exposure to the same viral inoculum. This protective phenotype was associated with the development and maintenance of neutralizing antibodies detected by CPE-VNT. Furthermore, neutralizing activity was maintained or increased in some animals after secondary exposure, suggesting effective activation of immunological memory responses.

It is important to highlight that neutralizing antibodies are considered one of the major correlates of protection against CHIKV infection, and previous studies have demonstrated that humoral immunity plays a critical role in restricting viral dissemination and preventing reinfection [24, 25]. In this context, although the increase in neutralizing antibody titers following homologous rechallenge did not reach statistical significance (*p* = 0.0625), the upward trend observed in some animals may indicate a biologically relevant recall response. This interpretation should be considered cautiously, but, together with the maintenance of neutralizing activity and the absence of detectable viremia after rechallenge, it supports the utility of this experimental platform for investigating protective immune responses and evaluating vaccine candidates. However, it is important to emphasize that additional immune mechanisms, such as cellular immunity, may also be involved in this process. Importantly, the demonstration of protection against viremia after rechallenge in immunocompetent mice infected with a contemporary Brazilian isolate adds further evidence that natural CHIKV infection can induce robust protective immunity against homologous viral challenge.

Despite the advances provided by the present model for the investigation of acute CHIKV infection and protective immunity, some limitations should be acknowledged. The experimental design focused predominantly on acute infection and homologous reinfection using a single viral strain. Additional studies evaluating longer follow-up periods, histopathological alterations, cytokine profiles, differential leukocyte counts, chronic inflammatory manifestations, and heterologous challenge between distinct CHIKV genotypes may further expand the applicability of the model. Moreover, although the model reproduced important aspects of acute infection, chronic arthralgia-associated mechanisms were not investigated in the present study and remain an important area for future investigation. Future studies should also compare murine and human neutralizing antibody responses using harmonized assays and comparable sampling time points.

In conclusion, we established a reproducible immunocompetent murine model of CHIKV infection using a contemporary Brazilian ECSA isolate capable of reproducing relevant virological, inflammatory, and immunological features of acute chikungunya disease. The model demonstrated productive systemic infection, tissue dissemination, local inflammatory manifestations, leukocyte alterations, and protection against detectable viremia following homologous rechallenge. These findings provide a relevant experimental platform for future studies investigating CHIKV pathogenesis, immune correlates of protection, antiviral therapies, and vaccine development strategies.

## Supplementary Information

Below is the link to the electronic supplementary material.

Supplementary figure 1 (PNG 206 KB)

Supplementary Material 1. The CHIKV35 virus was titrated and subsequently used to generate a standard curve for RT-qPCR-based quantification. The standardized RT-qPCR method was applied to quantify the genetic material present in animal samples during primary infection. Ct values were plotted as a function of log TCID50/mL. Regression analysis demonstrated linearity (R^2^ = 0.9995) with a slope of -3.207, corresponding to an amplification efficiency of 105.5%. Data points represent the mean of technical replicates (TIF 40.4 KB)

## Data Availability

Genome sequence of a chikungunya virus isolate was generated in this study and submited to GISAID. The GISAID numer deposit is cited in the text.
